# Ergonomic guidelines for the design interfaces of additive modules for manual wheelchairs: sagittal plane

**DOI:** 10.1038/s41598-023-39085-7

**Published:** 2023-07-25

**Authors:** Bartosz Wieczorek, Mateusz Kukla, Łukasz Warguła, Marcin Giedrowicz

**Affiliations:** 1 Faculty of Mechanical Engineering, Poznan University of Technology, Piotrowo 3 St., 424 BM, 61-139 Poznan, Poland; 2 Faculty of Architecture, Poznan University of Technology, Poznan, Poland

**Keywords:** Risk factors, Biomedical engineering, Mechanical engineering

## Abstract

When designing wheelchair propulsion systems operated with the upper limb, there is a noticeable lack of ergonomic analyses informing about the areas on the wheelchair frame where hand-operated controls can be installed. With that in mind, a research goal was set to measure the areas of human hand reach within the area defined by the structural elements of a manual wheelchair. An ergonomic analysis was performed on a group of ten patients representing 50% of anthropometric dimensions. Motion capture and image analysis software based on the openCV library were used for the measurement. The conducted research resulted in the development of a map of the hands range in the lateral plane of the wheelchair, parallel to the sagittal plane. In addition, the map was divided into three zones of hand reach, taking into account various levels of comfort of hand manipulation. The total hand reach area was 1269 mm long and 731 mm high, while the most comfortable manipulation area was 352 mm long and 649 mm high. The plotted hands reach areas act as a map informing the designer where on the sagittal plane additional accessories operated by the user can be installed.

## Introduction

The wheelchair, together with the human that operates it, creates an anthropotechnical system in which the wheelchair replaces the functions of the human body that are limited by disability, including the most important one i.e. the ability to move. For this purpose, the wheelchair is equipped with an electric or manual propulsion system. In the group of the manual propulsion systems, the most popular one is the push propulsion system, powered by the user's upper limbs^1–3^. The simple design of the pushrims drive translates into ease of use while increasing muscle effort. The simple design translates into failure-free operation and ease of use, but also a lot of effort, especially when using a wheelchair to climb up a hill^4,5^ or when moving in non-urban areas^6^.

**Figure 1:**
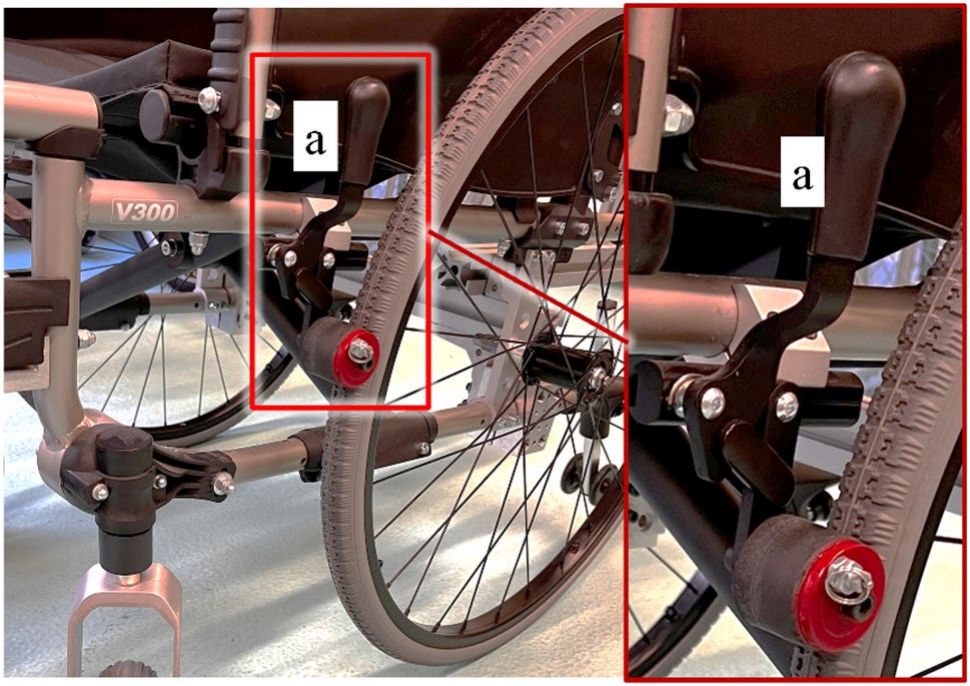
Prototype of the anti-rollback system mounted on a wheelchair with a pushrim propulsion with the position of the control lever marked (**a**).

The simple design and versatility of the manual propulsion system make it widely used. The manufacturers of wheelchairs, to eliminate their disadvantages and increase availability, introduce a number of modifications that increased efficiency. The modifications involve handrims structure changes^7^, as well as retrofitting individual wheelchairs with additional modules supporting the manual propulsion system^8,9^. Additional modules installed by the manufacturers are most often located within the rear wheels, on the side surface of the wheelchair frame and require additional handling by the user. The anti-rollback system, for example^8^ (Fig. 1), is mounted close to the rear wheel and the turn on/off lever (a) is located in a place where it may collide with the hand ending the cycle of propulsion^10–12^.

**Figure 2:**
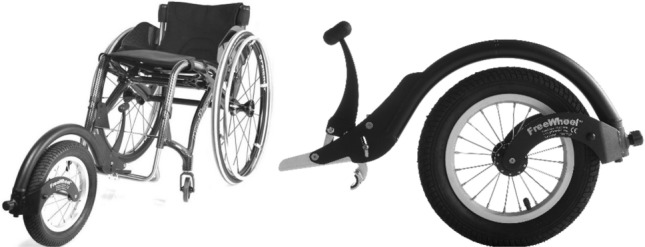
FreeWheel additional caster wheel module distributed by Spokz.

Other problems occur when using the FreeWheel module (Fig. 2). It is mounted in the front section of the wheelchair, so it does not collide with the hand pushing the pushrim. However, due to the location of the module, the user’s trunk must be bent for operation. For some wheelchair users, this is impossible due to the lack of trunk stabilisation resulting from paralysis of the back and abdominal muscles.

When analysing the market of additional modules, it was found that, depending on the function performed, they are mounted in different places on the frame of the wheelchair. Very often, their location is very close to the rotating, hand-pushed elements of the propulsion system. What is more, the wheelchair-mounted modules require handling that involves the human body kinematic chains of various lengths^13^ which, in extreme cases, may result in the loss of its stability due to the lack of stabilisation by the wheelchair seat^14^. Due to the above, it was determined that when designing additional modules, ergonomic aspects should be taken into account^15^. This is very important since one of the criteria for the mobility of a wheelchair is its adjustment to the user’s physical features^16^. Currently, anthropometric atlases^17^ are commonly used at the stage of design. However, there is a noticeable lack of criteria determining the comfort of using technical devices depending on the length of the kinematic chain of the human body used to operate it. The only available research on the comfort of use deals with the analysis of the influence of the seat position in relation to the push propulsion on the comfort of driving a wheelchair^18^. In addition, there is a noticeable lack of research linking the upper limbs reach with the positioning of the body in the wheelchair and the geometric features of the wheelchair frame. Among the available works, there are studies describing the impact of wheelchair configuration on its mobility^19^ or studies analysing the type and frequency of activities performed in a wheelchair^20^.

In connection with the above considerations, a research goal was set. The objective was to measure the areas within the reach of the human hand within the structural elements of the manual wheelchair used to attach accessories that increase the functionality of the wheelchair. The premise for undertaking the research was the hypothesis assuming the designation of several zones in which additional wheelchair accessories can be attached. Each of these zones will use the kinematic chain of the human body of different lengths. The benefit of the obtained results is the preparation of guidelines for wheelchair designers, informing about where to place the control interface of additional modules, and in which zones the manual operation of the control interface should be simplified.

## Method and materials

### Research procedure

**Figure 3:**
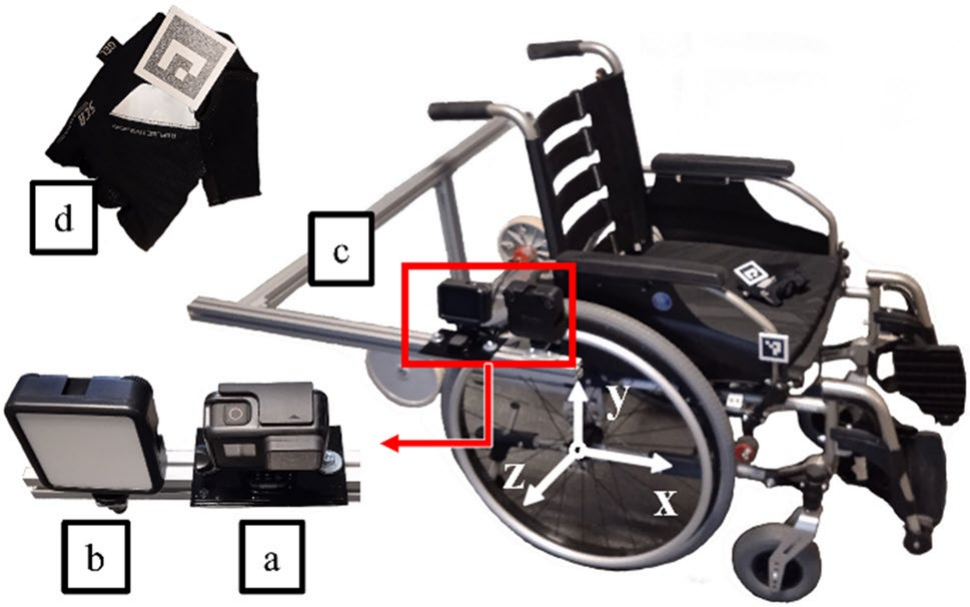
The wheelchair and the measuring equipment used during the research: (**a**) camera, (**b**) illuminating light, (**c**) boom, (**d**) AruCo marker.

The research tests were carried out in real conditions of wheelchair use. A semi-active Vermeiren v300 wheelchair equipped with a hand position recording system was used for the study (Fig. 3). Hand range analysis was limited to the observation of the marker placed on the glove (Fig. 3d). The measuring system used consisted of a GoPro HERO 7 camera (Fig. 3a) and an illuminating lamp (Fig. 3b) mounted on the boom (Fig. 3c) permanently fixed to the frame of the wheelchair. The camera records the image in 960p quality with a speed of 240 fps. The illuminating lamp emitted 200 to 1000 lm, depending on the intensity of the ambient light. AruCo codes were used as markers^21^, printed on 50 × 50 mm plates.

**Figure 4:**
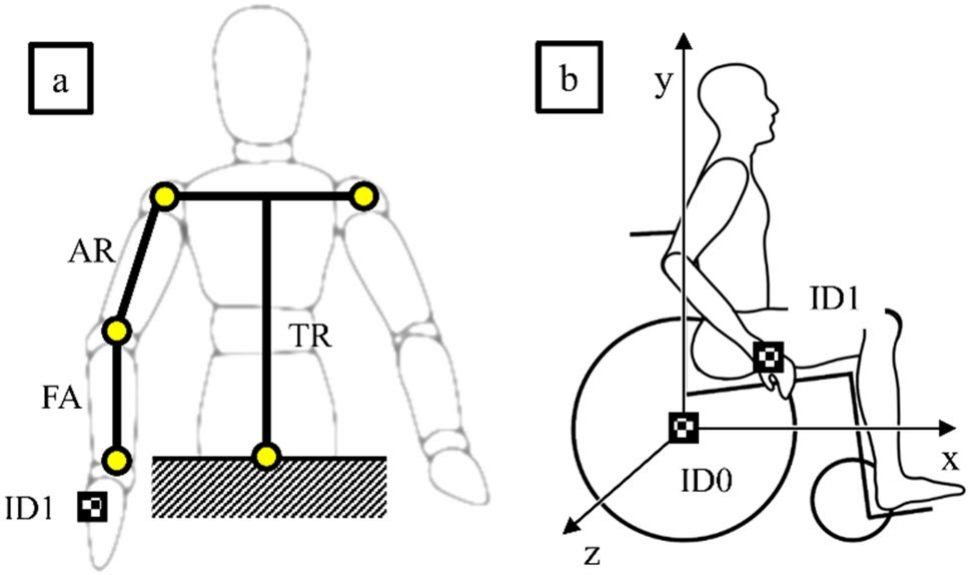
Diagram of the human body with the marked kinematic chain included in test (**a**) and of the patient in a wheelchair with the locations of the registered markers (**b**), where: *ID1* marker placed on the hand, *ID0* reference marker, *TR* trunk segment, *AR* arm segment, *FA* forearm segment.

The research procedure included the measurement of the position of the marker placed on the patient's hand (ID1) in relation to the stationary marker located in the wheelchair rear wheel axis of rotation (ID0) (Fig. 4). For the motion capture measurement, proprietary software was used that uses image processing of AruCo markers using OpenCV libraries that allow to determine their location in space relative to the immobile ID0 marker. It should be noted that other methods can be used to capture motion, e.g. using RGB-D Sensors^22^. Measurements were made in the sagittal plane^23^, which is parallel to the plane of the assumed XY datum. This approach is in line with the already developed two-dimensional wheelchair drive model^24^.

**Figure 5:**
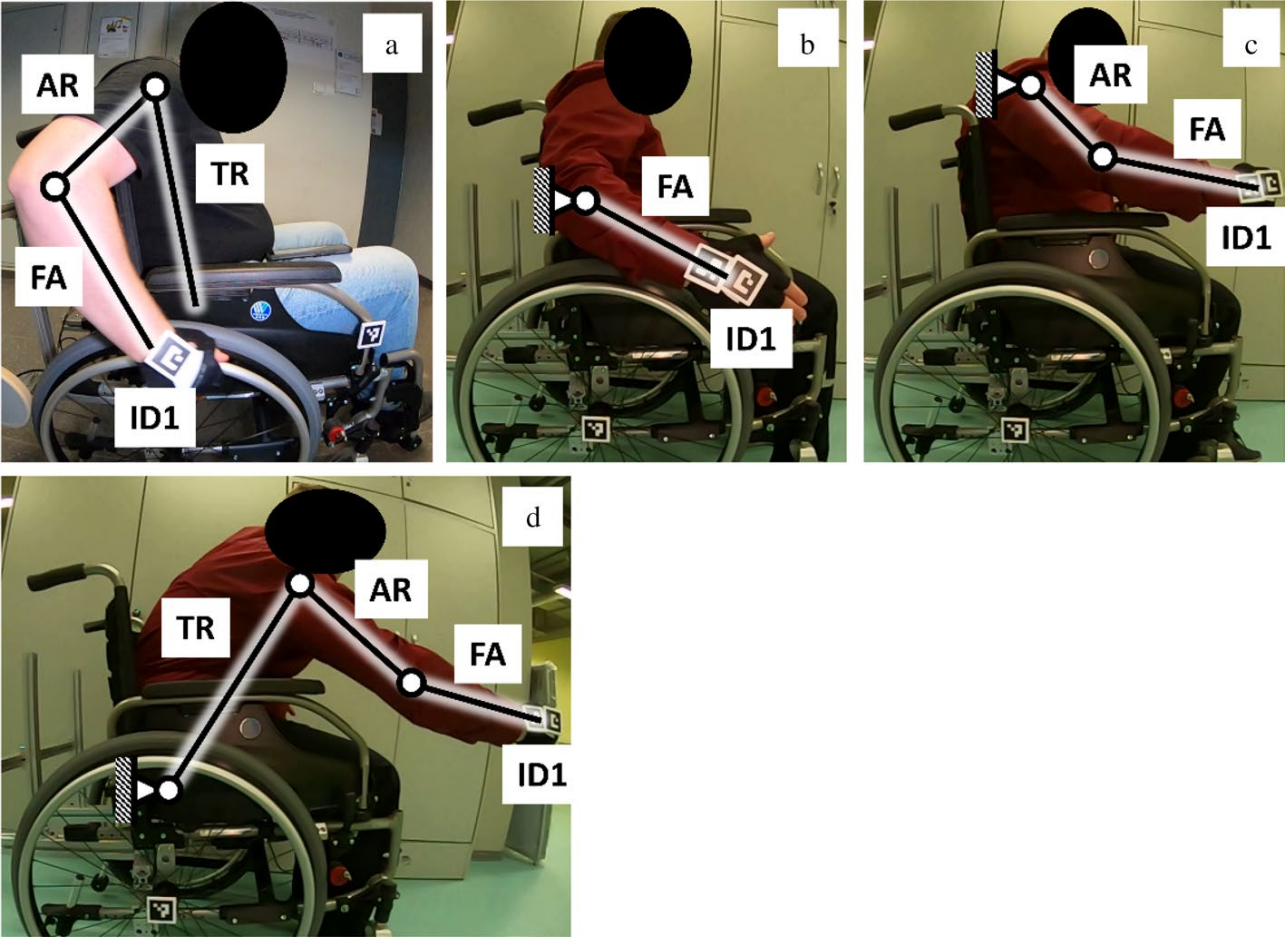
Diagram of the length of the human body kinematic chain when driving a wheelchair (AoP) (**a**), during manipulation of the upper limb in the area of comfort (AoC) (**b**), during manipulation of the upper limb in the area of approval (AoA) (**c**) and during manipulation of the upper limb in the area of risk (AoR) (**d**). Where: *ID1* observed marker, *FA* forearm, *AR* arm, *TR* trunk.

The research procedure includes the determination of four areas of hand manipulation with the use of motion capture measurement (Fig. 5). Each area was designated as the mean of the three measurement tests performed by each of the ten patients.

- The first area called the area of propulsion (AoP) defined the position of the hand while propelling the wheelchair (measured in real conditions during propelling the wheelchair).
- The second area called area of comfort (AoC) defined the free movement of the kinematic chain consisting of the forearm (FA), while the arm (AR) was stationary and directed downwards parallel to the trunk (TR) (measured under quasi-static conditions).
- The third area called the area of approval (AoA) defined the free movement of the kinematic chain consisting of the forearm (FA) and the arm (AR), while keeping the trunk (TR) stationary against the backrest of the wheelchair (measured under quasi-static conditions).
- The fourth area called the area of risk (AoR) defined the free movement of the kinematic chain consisting of the forearm (FA), the arm (AR) and the trunk (TR), while keeping the hip motionless on the seat (measured under quasi-static conditions).

The segmentation of the human body used in the study complies with the accepted biomechanical standards^25,26^, and the adopted location of the ID1 marker on the hand complies with the anthropometric guidelines^16,27^.

### Tested men

Ten patients took part in the tests (Table 1) and they were subdivided by height, weight, age and wheelchair experience. Experience was determined on the basis of seniority in using the wheelchair. The patients participating in the tests reflected the 50th percentile of anthropometric dimensions according to the European standard "Basic list of definitions of human body dimensions for technical design" (EN 979). The decisive criterion for the patient's participation in the study was his anthropometric dimensions. Efforts were made to select the measurement group so that it represented the same percentile (50th percentile) of anthropometric dimensions. In addition, attention was paid to the similar age of the user and physical condition. This allowed us to assume that their ranges of limit joint deflections are similar. The research and experimental protocols has been positively evaluated by Bioethical Commission at the Karol Marcinkowski Medical University in Poznań Poland, Resolution No. 1100/16 of 10 November 2016, under the guidance of Prof. MD P. Chęciński for the research team led by Wieczorek B. The authors obtained the written informed consent of the examined person for the publication of research results with her participation. The data was presented in such a way as to ensure her complete anonymity. The measurement method and data acquisition were carried out in accordance with the directives of the Bioethics Commission at the Karol Marcinkowski Medical University in Poznań Poland, which are in line with the guidelines Declarations of Helsinki.

**Table 1:** Comparison of anthropometric features and the level of experience in wheelchair operation of the test subjects.

	Height (cm)	Weight (kg)	Age (years)	Experience (–)
Subject MK	183	90	32	●●●●●
Subject MKA	179	88	33	●●●●●
Subject BW	175	110	31	●●●●○
Subject BWA	178	96	30	●●●○○
Subject LWA	171	93	33	●●●○○
Subject LW	173	87	32	●●●●○
Subject DRA	169	72	30	●●●○○
Subject DR	174	81	35	●●●●○
Subject MKB	180	74	36	●●●○○
Subject MKC	174	72	36	●○○○○
AVG	176 ± 3	86 ± 9	33 ± 2	–

Mean values determined with the 95% confidence interval (p = 0.05).

### Data processing procedure

**Figure 6:**
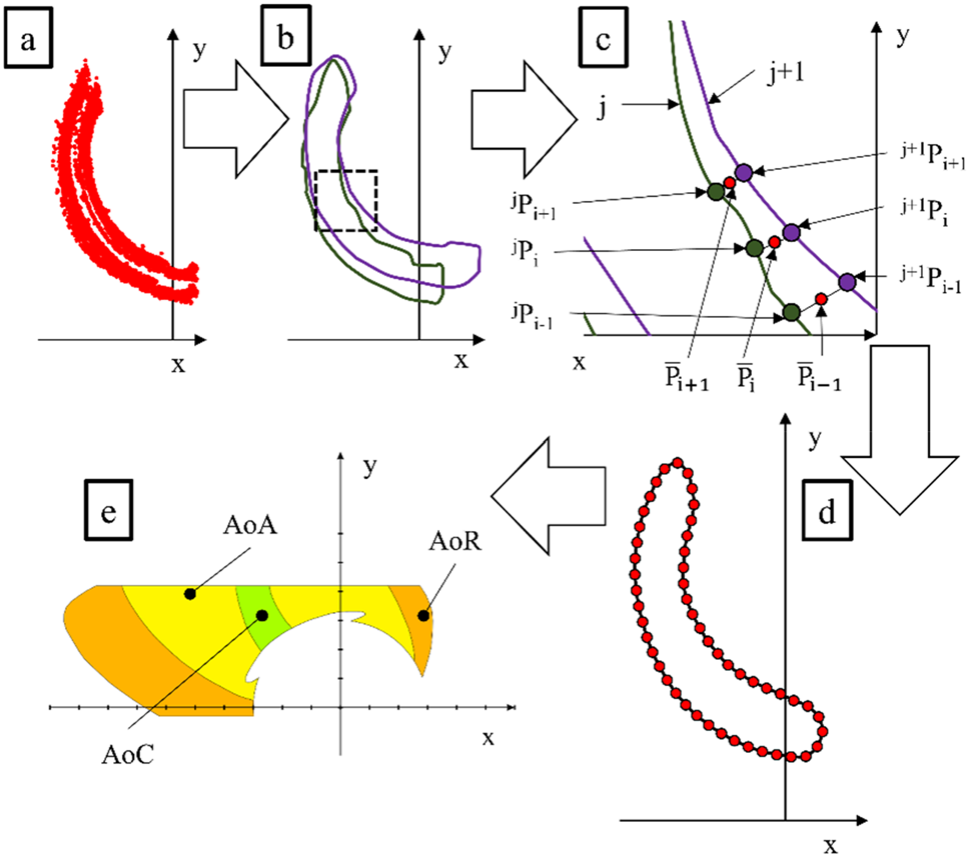
Data processing algorithm procedure from motion capture measurement, where (**a**) cloud of analyzed points, (**b**) outline of points generated by the alpha shape algorithm, (**c**) a scheme for determining the points of the average outline, (**d**) scheme for determining the average outline with marked forming points, (**e**) an example of palm range areas determined on the basis of sums and differences of determined average palm range contours, *x* horizontal axis, *y* vertical axis, *P* the point located on the loop describing the point cloud area, *j* index for numbering the analysed loops, *i* index for numbering the points on the loop, ${\overline{\text{P}}}$ the point on the averaged loop describing the hand range, *AoP* the area of presence, *AoC* the area of comfort, *AoA* the area of approval, *AoR* the area of risk.

The data processing algorithm used to define the areas of hand manipulation by the manual wheelchair user in the sagittal plane involved five steps (Fig. 6). In the first step (Fig. 6a), the image recorded with the camera was converted into a set of points defined by two coordinates, horizontal “x” and vertical “y”. For this purpose, proprietary software based on the OpenCV library was used^28^. The software recognised the position of marker ID1 relative to marker ID0. Then, using the alpha shape algorithm^29,30^, a designated closed-loop cloud was determined (Fig. 6b). When using the algorithm, the alpha coefficient ranged from 0.7 to 0.9, depending on the obtained point cloud density. Because after using the alpha shape algorithm, each loop consisted of a different number of points, it was necessary to uniform their number. Therefore, using Rihnoceros 3D software, each loop was converted so that it would be described with a hundred of ^j^P_i_ points. Where “j” is the index of the considered loop and “i” is the index of the considered point in the loop (Fig. 6c). Rhinoceros software with Grasshopper module was used for this activity. The next step was to search for all the designated loops of points between which the distance was the shortest. Using the points determined this way, the mean point ${\overline{\text{P}}}$_i_, was calculated, which defined the averaged contour of the analysed area (Fig. 6d). The last stage was plotting the defined averaged areas on a common graph (Fig. 6e).

## Results

**Figure 7:**
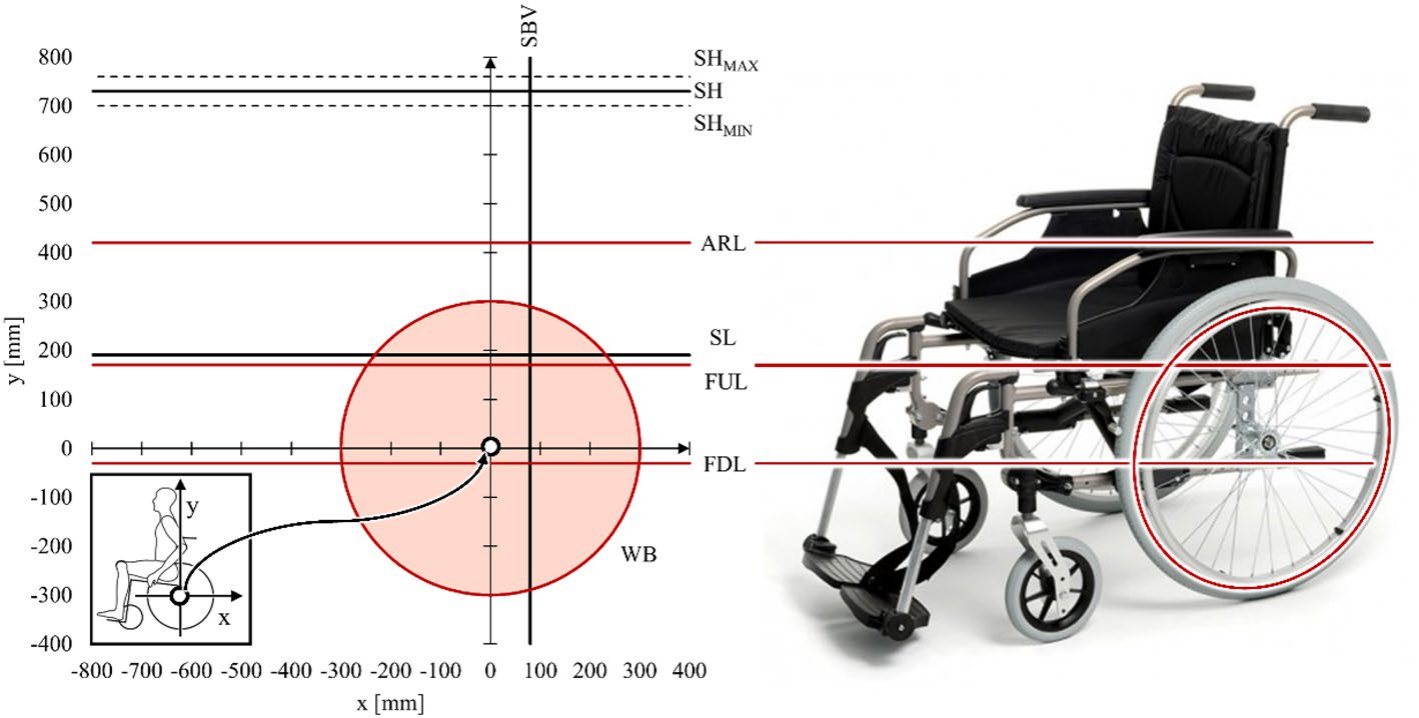
Geometric features diagram on the sagittal plane of the analysed human–wheelchair system. Where: S*H* shoulder level for the trunk resting on the backrest of the wheelchair, *SH*_*MIN*_ minimum shoulder level for the trunk resting on the backrest of the wheelchair, *SH*_*MAX*_ maximum shoulder level for the trunk resting on the backrest of the wheelchair, *SBV* seat backrest vertical angle, *SL* seat level, *ARL* armrests height, *FUL* the height of the upper part of the wheelchair frame, *FDL* the height of the lower part of the wheelchair frame, *WB* the contour outline of the rear wheel.

At the beginning of the ergonomic analysis, characteristic geometric features were determined for the considered anthropotechnical system between the man and the wheelchair (Fig. 7) (Table 2). The geometrical features defining the possible direction of extension and modification of the wheelchair are defined by the lower level of the frame (FDL), the upper level of the frame (FUL) and the level of the armrests (ARL). The levels defining the position of the human body in the wheelchair are described by the seat level (SL), the seat backrest vertical angle (SBV), the rear wheel spinning area (WB) and the shoulder height (SH). In this comparison, the shoulder level is the only anthropometric dimension determined experimentally, therefore the confidence interval (SH_MIN_ and SH_MAX_) was determined for the measurement sample N = 10 and the confidence level p = 0.05.

**Table 2:** Parametric equations defining the geometrical features on the sagittal plane of the analysed human–wheelchair system.

Geometric feature	Parametric equations		Geometric feature	Parametric equations	
	X (mm)	y (mm)		x (mm)	y (mm)
FDL	n/a	$y=-30$	SL	n/a	$y=190$
FUL	n/a	$y=170$	SH	n/a	$y=730\pm 30$
ARL	n/a	$y=420$	SBV	$x=80$	n/a
WB	$x=300\cdot \mathrm{sin}(t)$	$y=300\cdot\mathrm{cos}(t)$			

Where: *SH* shoulder level for the trunk resting on the backrest of the wheelchair, *SH*_*MIN*_ minimum shoulder level for the trunk resting on the backrest of the wheelchair, *SH*_*MAX*_ maximum shoulder level for the trunk resting on the backrest of the wheelchair, *SBV* seat backrest vertical angle, *SL* seat level, *ARL* armrests height, *FUL* the height of the upper part of the wheelchair frame, *FDL* the height of the lower part of the wheelchair frame, *WB* the contour outline of the rear wheel.

The geometrical features SBV and SL define the position of the seat in relation to the axis of rotation of the drive wheel. They are often selected individually by each user^1,31^. Therefore, in the adopted ergonomic model, they can be freely modified. However, when modifying the features (in relation to the data given in Table 2), the designated areas of reach of the hand of a person sitting in a wheelchair should be properly shifted vertically and horizontally. These shifts should be the same as the changes in the seat position in relation to the data adopted in the work. To determine the geometrical features FDL, FUL and ARL defining the dimensions of the frame of the wheelchair, the dimensions of the frame of a semi-active wheelchair popular in Europe were used. These features can also be freely changed depending on the adopted type of wheelchair.

**Figure 8:**
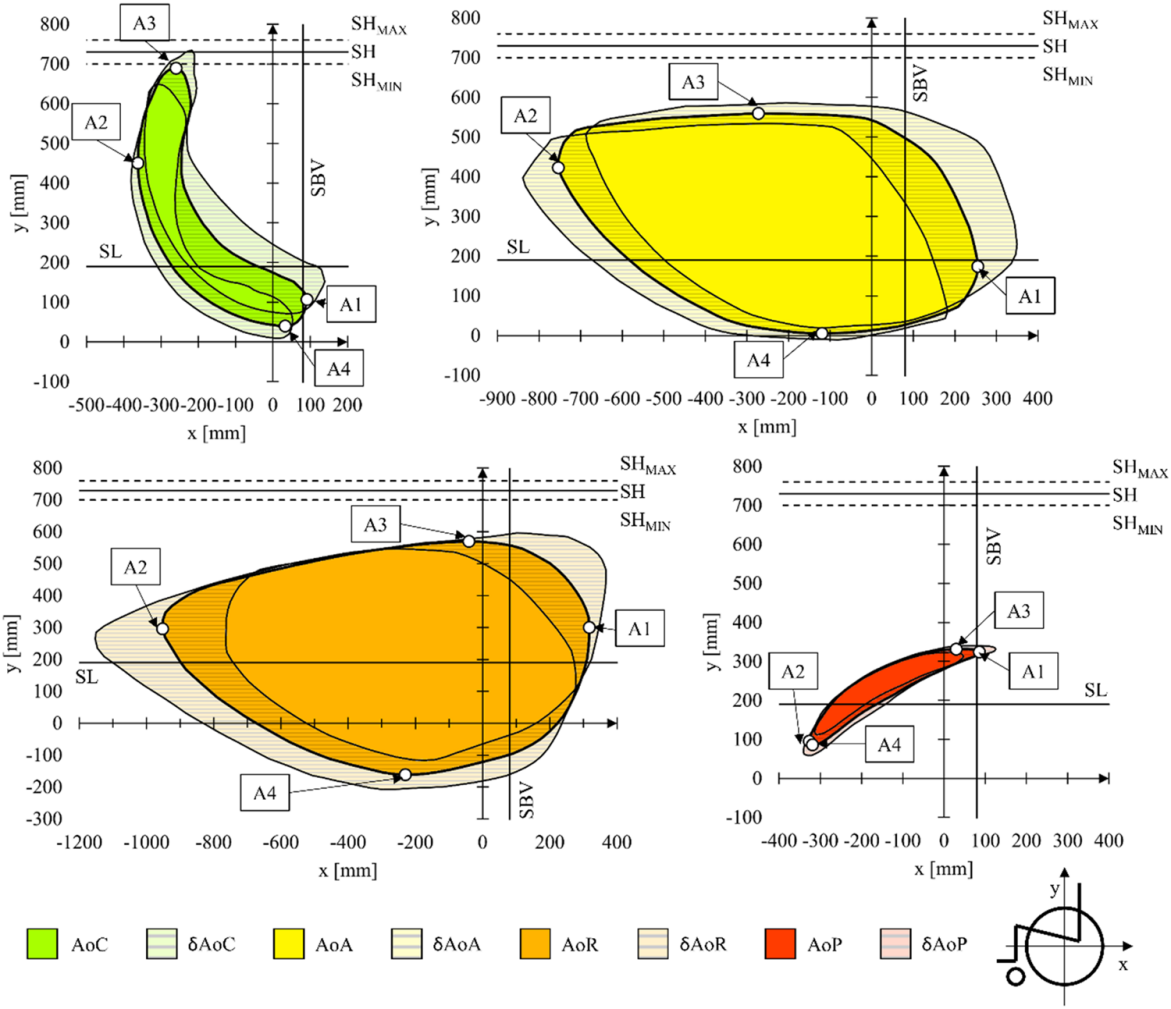
Reach areas of a human hand sitting in a wheelchair depending on the mobility of the analysed kinematic chain. Where: *SH* shoulder level for the trunk resting on the backrest of the wheelchair, *SH*_*MIN*_ minimum shoulder level for the trunk resting on the backrest of the wheelchair, *SH*_*MAX*_ maximum shoulder level for the trunk resting on the backrest of the wheelchair, *SBV* backrest vertical angle, *SL* seat level, *A1–A4* control points defining the contours of the designated areas, *AoC* the area of comfort, *δAoC* dimension deviation for the area of comfort, *AoA* the area of approval, *δAoA* dimension deviation for the area of comfort, *AoR* the area of risk, *δAoR* dimension deviation for the area of risk, *AoP* the area of propulsion, *δAoP* dimension deviation for the area of propulsion.

Having defined permanent geometrical features of the man and the wheelchair, the areas of reach of the hand of the person sitting in a wheelchair were determined (Fig. 8). These ranges were determined by subdividing them into four areas. The designated areas are the average value of 30 tests (N = 30) for which the confidence interval was determined for the confidence level of 95% (p = 0.05). The additional materials (Supplementary Table 1–4, Supplementary Fig. 1–4) contain a complete set of 100 points describing each area. Additionally, four control points were determined for each of the areas (Table 3). The points illustrate the height and width of each field and serve as control points that can be used to shift areas in the sagittal plane when analysing wheelchair types other than the ones used in the tests.

**Table 3:** List of control points in the designated areas of hand reach.

Point ID	x (mm)	y (mm)	δx (mm)	δy (mm)	Δ (mm)	n (n/a)	p (n/a)
Area of comfort (AoC)							
A1	92	107	42	33	108	30	0.05
A2	−362	450	18	45	97	30	0.05
A3	−260	689	33	43	109	30	0.05
A4	33	40	21	31	76	30	0.05
Area of approval AoA							
A1	255	174	92	32	109	30	0.05
A2	−754	423	85	25	97	30	0.05
A3	−272	559	65	27	109	30	0.05
A4	−120	6	18	14	76	30	0.05
Area of risk (AoR)							
A1	317	300	46	103	225	30	0.05
A2	−952	296	194	60	406	30	0.05
A3	−41	570	141	26	288	30	0.05
A4	−230	−161	31	46	111	30	0.05
Area of comfort (AoC)							
A1	85	324	40	8	81	30	0.05
A2	−327	95	14	22	52	30	0.05
A3	30	331	42	9	86	30	0.05
A4	−319	86	16	26	61	30	0.05

Where: *x* *and* *y* location of the control point on the sagittal plane, *δx* confidence interval of the control point location on the horizontal axis, *δy* confidence interval of the control point location on the vertical axis, *Δ* the distance between the extreme positions of the control point, *n* the size of the sample used to calculate the control point position, *p* adopted confidence level.

To interpret the obtained results, the lengths of the confidence intervals were calculated for the adopted control points Δ (Eq. 1).

1 $$\Delta =\sqrt{{\left[\left(x+\delta x\right)-\left(x-\delta x\right)\right]}^{2}+{\left[\left(y+\delta y\right)-\left(y-\delta y\right)\right]}^{2}}$$

When analysing the results, it was found that AoR is the largest area of hand manipulation. This area is 1269 mm long and 731 mm high. However, the greatest values of the confidence interval Δ were measured for the area located between extreme values of control points. These distances were on average 258 mm. For comparison, the average distance Δvalues for the AoA and AoC areas were 140 mm and 98 mm, respectively. The phenomenon of such differences in the lengths of the confidence intervals in individual areas is the effect of a different number of kinematic chains body segments^32,33^ used during their determination. In the case of the AoR area, significant differences between the mean value of the area and its dimensions taking into account the confidence interval result from the share of the trunk in the kinematic chain used. Each tested patient had different mobility of trunk, so the greatest differences in hand reach were examined^34^. Consequently, the AoR has been classified as ergonomically risky to install the accessory interface of the manual wheelchair. The ergonomic risk of installing the interface in this area results from the inability to clearly determine the limit inclinations of the trunk at which the user is not at risk of falling from a wheelchair. Differences in the trunk angle between individual patients result not only from physical abilities^35^ but also from wheelchair accessories such as seat belts^36^.

The AoA area in which the trunk was resting on the seat was 1009 mm in length and 553 mm in height, so the dimensions slightly smaller than those in the AoR area. However, in this case, much shorter lengths Δ were noted between the extreme positions of the control points and the control points with consideration of the confidence interval. This translates into greater precision in defining the reach of the hand. Additionally, a person who is physically disabled using a kinematic chain consisting only of the upper arm and forearm has the trunk stabilised. As a result, the risk of falling when tilting the trunk is minimised^37^.

The AoC area has been defined as the most comfortable one because it involves the shortest kinematic chain including the forearm only. As a result, the person using the wheelchair is subjected to the least physical effort^38^. Despite these advantages, this area had the smallest surface area. Its length was 352 mm and the height was 649 mm.

The last AoP examined described the area of hand manipulation while propelling the wheelchair. This area defines the place that should be free from additional accessories and should allow the user to freely manipulate it while propelling the wheelchair. The smallest length Δ dispersion was observed for the AoP area between the extreme positions of the control points and the control points with consideration of the confidence interval. The length of the AoP area was 412 mm and its height was 245 mm. It should be noted that the designated area of AoP coincides with the trajectories of movement of the hand pushing the handrim^38–40^.

## Discussion

**Figure 9:**
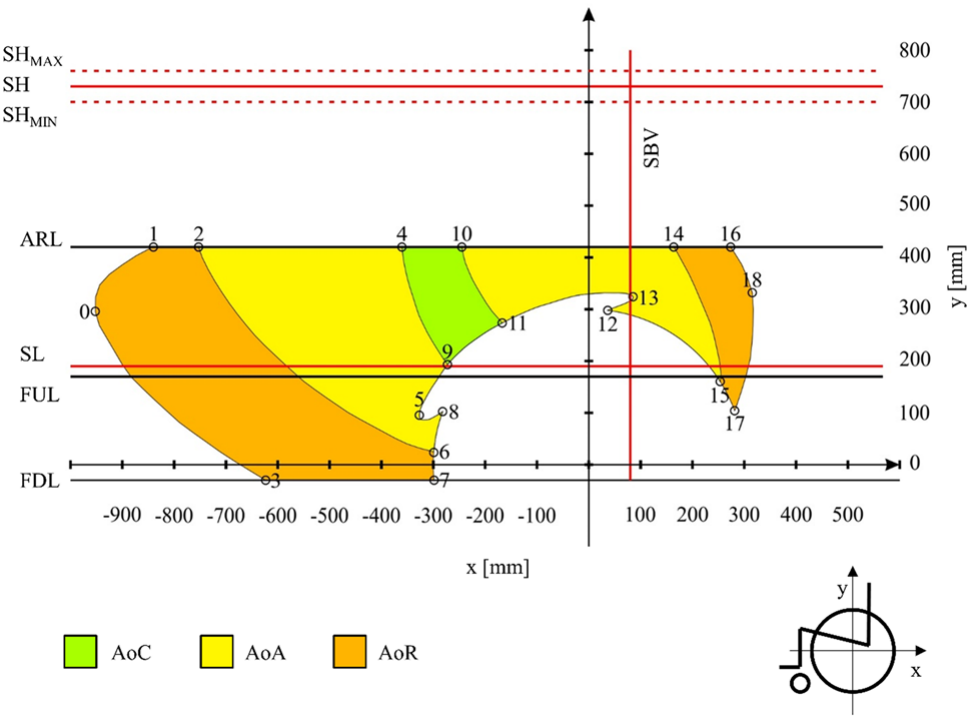
Areas of reach of the hand, taking into account the geometrical features of the wheelchair. Where: *SH* shoulder level for the trunk resting on the backrest of the wheelchair, *SH*_*MIN*_ minimum shoulder level for the trunk resting on the backrest of the wheelchair, *SH*_*MAX*_ maximum shoulder level for the trunk resting on the backrest of the wheelchair, *SBV* seat backrest vertical angle, *SL* seat level, *ARL* armrests height, *FUL* the height of the upper part of the wheelchair frame, *FDL* the height of the lower part of the wheelchair frame, *AoC* the area of comfort, *AoA* the area of approval, *AoR* the area of risk.

According to the methodological assumption, the defined permanent geometrical features of the human body and the wheelchair, as well as the measured areas of reach of the hand were superimposed. As a result, three areas of reach of the hand were obtained within the lower edge of the frame and the armrest of the wheelchair (Fig. 9). In order to implement the results in design problems, these areas were defined using a set of points enabling their approximate plotting (Table 4).

**Table 4:** List of points describing the areas of reach of the hand, taking into account the geometrical features of the wheelchair and the areas reserved for the use of the manual propulsion system.

Point ID	x (mm)	Y (mm)	Point ID	x (mm)	y (mm)	Point ID	x (mm)	y (mm)
0	−951	296	7	−298	−30	14	164	420
1	−840	420	8	−282	102	15	253	160
2	−753	420	9	−273	193	16	274	420
3	−623	−30	10	−245	420	17	281	104
4	−361	420	11	−167	274	18	317	300
5	−327	95	12	37	298			
6	−299	25	13	85	324			

Where *x* position of a point on the horizontal axis, *y* position of a point on the vertical axis.

The designated areas illustrates the three comfort zones for hand manipulation, located within three levels defined by the geometric features of the wheelchair. The area between the FUL and FDL levels illustrate the places on the wheelchair frame that do not change their position in relation to the drive wheel. The area between the ARL and FUL levels is where the armrests are located. This area was separated because during the use of the wheelchair (e.g. for dismounting^41^) they are disassembled. Therefore, when planning to install additional accessories on these elements, one should take into account the need for its occasional disassembly. The performed subtraction of the previously determined areas showed that the most comfortable AoC in terms of safety and effort reduction is significantly reduced by the area reserved for the spinning wheel WB (Fig. 7) and the area in which the hand that pushes the handrim (AoP). After considering these areas, the AoC area is 194 mm wide and 227 mm high.

The main determinant of the shape of the developed areas (Fig. 9) is the length of the trunk, arm and forearm. Comparing the obtained results, a similarity with the data available in anthropometric atlases was noticed. An example of this is point 2 (Table 4, Fig. 9) in which the torso was leaning against the backrest (SBV) and the upper limb was straightened and in a position close to horizontal. For such a system, the value on the x-axis representing the length of the arm and forearm is 753 mm, this value coincides with the data from anthropometric atlases. According to which the length of the upper limb measured from the axis of rotation of the shoulder joint to the center of the clenched fist is 783 mm^41^, 765 mm^42^ and 743 mm^43^ for a 50th percentile male. The same relationship can be observed for point 4, for which the torso was supported with the forearm horizontally set at an angle of 90 degrees to the arm. For this configuration, taking into account the offset of the wheelchair backrest (SVB), the x-value (corresponding approximately to the length of the forearm) was measured, which is 441 mm. For comparison, an anthropometric atlas^42^ gives the forearm length of a 50th percentile male as 472 mm. The high convergence of the results describing the anthropometric dimensions measured during the experiment with the results published in anthropometric atlases confirms the correctness and reference to reality of the developed areas within the reach of the hand. In addition, analyzing the available literature data, a convergence of the length of the upper limb measured in the article with the data used as input data in other publications was noticed. An example may be a publication dealing with the problem of developing a scalable musculoskeletal model^44^ in which the patient corresponding to the 50th percentile of anthropometric dimensions had an upper limb 784 mm long, which is a 31 mm difference compared to the results obtained in the study.

Referring to the vertical and horizontal limitations marked in Fig. 9: FDL, FUL, ARL, SL, SH and SVB and their location in space, it should be noted that these are constant values that do not depend on the position of the human body, only on the design features of the wheelchair. They result from the geometric features of the wheelchair structure used. These are shaved available data that can be downloaded from the website of the manufacturer of the tested wheelchair. In addition, these data are available in the instruction manual supplied with the wheelchair. This approach makes the results valid for the cross-frame semi-active wheelchair group. This introduces a certain limitation and inconsistency of the map developed in Fig. 9 with wheelchairs from the group of active wheelchairs with a fixed frame. However, for these wheelchairs, it is possible to implement designated hand-reach areas that do not take into account the design features of the wheelchair (Fig. 8, Table 3).

## Conclusions

The plotted AoC, AoA and AoR areas (Fig. 9) can be used as a map informing the designer where on the sagittal plane additional accessories operated by the upper limb of the user can be installed. The authors proposed a subdivision into three areas, depending on the range of the kinematic chain used. The AoC area was classified as the most comfortable because the movement of the hand within it translates into the least effort. The AoR area illustrated the limits of hand ranges of a man sitting in a wheelchair. The area was classified as the least comfortable because manipulation within its limits may increase the effort and the risk of falling off the wheelchair. The classification of areas presented in the work confirms the research hypothesis about the possibility of dividing the reach of the hand into different areas where the operation of the interface requires different lengths of the kinematic chain.

Using the developed ergonomic analysis, the designer can design additional accessories on the frame of the wheelchair. The accessories should be arranged according to the frequency of operation and the complexity of manipulations performed by the hand. Separated areas are helpful when arranging accessories. The accessories used frequently or requiring the use of increased physical force should be located within the AoC or AoA areas. Whereas the accessories that are used sporadically or do not require the use of significant force may be located within the AoR area.

When determining the AoR and AoE area contours, patients were not asked to raise the forearm vertically upward because the determined hand reach areas extend far beyond the designated levels of wheelchair geometric features. Such limitation of the entire limb range of motion did not affect the research goal and allowed us to bring the focal length of the camera closer to the tested object. Zooming in the focal length increased the accuracy of the measurement.

The conducted research supplements the deficiencies in anthropometric measurements, most of which focus on the reach of the hand of a person that is physically fit. In addition, the ergonomic analyses currently available are of generic nature and require further processing for implementation in the wheelchair design process. The developed map of hand movement ranges during manipulation within the wheelchair frame is equivalent of the available analyses of the geometry of the human workplace^45^ that currently the basis for the development of standards certifying offices, schools, etc.

The results presented in the study show only the areas lying on the sagittal plane. This plane was chosen due to the fact that most of the additional accessories, such as the parking brake, are mounted on this plane. In further works, the authors plan to examine the hand reach area in the front and rear planes of the wheelchair, parallel to the head plane.

When subdividing the hand reach areas, a classification based only on the length of the kinematic chain was used. Therefore, in further ergonomic analyses, it is possible to link the position of the hand to the measurement of the EMG signal measuring muscle activity. This will allow for a new subdivision of the hand reach into areas according to the different muscle activities.

## Supplementary Information

Supplementary Information.

## Data Availability

The datasets used and/or analysed during the current study available from the corresponding author on reasonable request.
