# Supplementary material for: Ergonomic guidelines for the design interfaces of additive modules for manual wheelchairs: sagittal plane
Source: Sci Rep. 2023 Jul 25;13:11993. doi: 10.1038/s41598-023-39085-7 (PMC10368659; doi:10.1038/s41598-023-39085-7)
Supplement: Supplementary file 1 — Supplementary Information. [file 41598_2023_39085_MOESM1_ESM.docx]

**Supplementary materials**

Table 1. List of points in the area of comfort (AoC). Where: x and y - location of the control point on the sagittal plane, δx - confidence interval of the control point location on the horizontal axis, δy - confidence interval of the control point location on the vertical axis, n - the size of the sample used to calculate the control point position, p - adopted confidence level.

| x | y | δx | δy | n | p |
| --- | --- | --- | --- | --- | --- |
| *[mm]* | *[mm]* | *[mm]* | *[mm]* | *n/a* | *n/a* |
| 14 | 41 | 21 | 30 | 30 | 0.05 |
| -4 | 43 | 21 | 30 | 30 | 0.05 |
| -22 | 46 | 21 | 29 | 30 | 0.05 |
| -41 | 49 | 20 | 29 | 30 | 0.05 |
| -59 | 53 | 20 | 28 | 30 | 0.05 |
| -76 | 59 | 20 | 27 | 30 | 0.05 |
| -94 | 65 | 20 | 27 | 30 | 0.05 |
| -111 | 71 | 20 | 27 | 30 | 0.05 |
| -128 | 79 | 20 | 27 | 30 | 0.05 |
| -145 | 87 | 20 | 27 | 30 | 0.05 |
| -161 | 96 | 20 | 28 | 30 | 0.05 |
| -177 | 106 | 20 | 29 | 30 | 0.05 |
| -192 | 116 | 20 | 29 | 30 | 0.05 |
| -207 | 127 | 20 | 30 | 30 | 0.05 |
| -222 | 139 | 20 | 30 | 30 | 0.05 |
| -235 | 151 | 19 | 31 | 30 | 0.05 |
| -248 | 164 | 18 | 32 | 30 | 0.05 |
| -261 | 178 | 17 | 32 | 30 | 0.05 |
| -272 | 193 | 17 | 32 | 30 | 0.05 |
| -283 | 208 | 17 | 33 | 30 | 0.05 |
| -293 | 224 | 17 | 34 | 30 | 0.05 |
| -303 | 239 | 17 | 34 | 30 | 0.05 |
| -312 | 256 | 17 | 35 | 30 | 0.05 |
| -320 | 272 | 17 | 36 | 30 | 0.05 |
| -328 | 289 | 17 | 37 | 30 | 0.05 |
| -334 | 306 | 16 | 38 | 30 | 0.05 |
| -339 | 324 | 16 | 39 | 30 | 0.05 |
| -345 | 342 | 15 | 40 | 30 | 0.05 |
| -350 | 359 | 15 | 41 | 30 | 0.05 |
| -354 | 377 | 16 | 42 | 30 | 0.05 |
| -358 | 395 | 17 | 42 | 30 | 0.05 |
| -360 | 414 | 18 | 43 | 30 | 0.05 |
| -362 | 432 | 17 | 44 | 30 | 0.05 |
| -362 | 450 | 18 | 45 | 30 | 0.05 |
| -362 | 469 | 17 | 46 | 30 | 0.05 |
| -361 | 487 | 17 | 47 | 30 | 0.05 |
| -358 | 506 | 17 | 48 | 30 | 0.05 |
| -356 | 524 | 16 | 49 | 30 | 0.05 |
| -352 | 542 | 15 | 50 | 30 | 0.05 |
| -348 | 560 | 14 | 50 | 30 | 0.05 |
| -343 | 577 | 15 | 51 | 30 | 0.05 |
| -336 | 594 | 16 | 52 | 30 | 0.05 |
| -328 | 611 | 19 | 52 | 30 | 0.05 |
| -320 | 627 | 22 | 50 | 30 | 0.05 |
| -313 | 644 | 26 | 49 | 30 | 0.05 |
| -305 | 659 | 31 | 47 | 30 | 0.05 |
| -296 | 672 | 36 | 41 | 30 | 0.05 |
| -285 | 681 | 38 | 38 | 30 | 0.05 |
| -273 | 689 | 36 | 39 | 30 | 0.05 |
| -260 | 689 | 33 | 43 | 30 | 0.05 |
| -247 | 685 | 30 | 50 | 30 | 0.05 |
| -237 | 671 | 27 | 52 | 30 | 0.05 |
| -231 | 655 | 21 | 52 | 30 | 0.05 |
| -226 | 639 | 14 | 52 | 30 | 0.05 |
| -223 | 622 | 12 | 52 | 30 | 0.05 |
| -220 | 604 | 13 | 53 | 30 | 0.05 |
| -220 | 586 | 15 | 53 | 30 | 0.05 |
| -225 | 569 | 18 | 53 | 30 | 0.05 |
| -229 | 551 | 18 | 53 | 30 | 0.05 |
| -235 | 534 | 16 | 54 | 30 | 0.05 |
| -239 | 516 | 15 | 55 | 30 | 0.05 |
| -242 | 498 | 13 | 56 | 30 | 0.05 |
| -244 | 480 | 13 | 57 | 30 | 0.05 |
| -245 | 462 | 14 | 57 | 30 | 0.05 |
| -246 | 444 | 15 | 58 | 30 | 0.05 |
| -246 | 426 | 17 | 58 | 30 | 0.05 |
| -243 | 408 | 19 | 60 | 30 | 0.05 |
| -239 | 391 | 24 | 61 | 30 | 0.05 |
| -232 | 374 | 28 | 62 | 30 | 0.05 |
| -225 | 358 | 32 | 62 | 30 | 0.05 |
| -216 | 342 | 36 | 62 | 30 | 0.05 |
| -207 | 326 | 40 | 62 | 30 | 0.05 |
| -197 | 311 | 43 | 63 | 30 | 0.05 |
| -186 | 296 | 47 | 62 | 30 | 0.05 |
| -174 | 282 | 50 | 62 | 30 | 0.05 |
| -162 | 268 | 52 | 61 | 30 | 0.05 |
| -150 | 255 | 55 | 60 | 30 | 0.05 |
| -137 | 243 | 58 | 58 | 30 | 0.05 |
| -122 | 232 | 61 | 54 | 30 | 0.05 |
| -108 | 223 | 63 | 51 | 30 | 0.05 |
| -92 | 214 | 65 | 49 | 30 | 0.05 |
| -76 | 206 | 66 | 46 | 30 | 0.05 |
| -59 | 198 | 67 | 43 | 30 | 0.05 |
| -43 | 191 | 68 | 40 | 30 | 0.05 |
| -26 | 185 | 69 | 37 | 30 | 0.05 |
| -9 | 179 | 70 | 34 | 30 | 0.05 |
| 7 | 172 | 71 | 33 | 30 | 0.05 |
| 23 | 164 | 74 | 33 | 30 | 0.05 |
| 39 | 158 | 75 | 33 | 30 | 0.05 |
| 56 | 152 | 75 | 31 | 30 | 0.05 |
| 67 | 141 | 67 | 28 | 30 | 0.05 |
| 78 | 131 | 59 | 28 | 30 | 0.05 |
| 87 | 121 | 52 | 31 | 30 | 0.05 |
| 92 | 107 | 42 | 33 | 30 | 0.05 |
| 88 | 90 | 36 | 33 | 30 | 0.05 |
| 83 | 74 | 31 | 31 | 30 | 0.05 |
| 75 | 61 | 26 | 30 | 30 | 0.05 |
| 65 | 50 | 22 | 31 | 30 | 0.05 |
| 50 | 42 | 22 | 32 | 30 | 0.05 |
| 33 | 40 | 22 | 31 | 30 | 0.05 |
| 14 | 41 | 21 | 30 | 30 | 0.05 |
| 14 | 41 | 21 | 30 | 30 | 0.05 |
| -4 | 43 | 21 | 30 | 30 | 0.05 |
| -22 | 46 | 21 | 29 | 30 | 0.05 |
| -41 | 49 | 20 | 29 | 30 | 0.05 |
| -59 | 53 | 20 | 28 | 30 | 0.05 |
| -76 | 59 | 20 | 27 | 30 | 0.05 |
| -94 | 65 | 20 | 27 | 30 | 0.05 |
| -111 | 71 | 20 | 27 | 30 | 0.05 |
| -128 | 79 | 20 | 27 | 30 | 0.05 |
| -145 | 87 | 20 | 27 | 30 | 0.05 |
| -161 | 96 | 20 | 28 | 30 | 0.05 |
| -177 | 106 | 20 | 29 | 30 | 0.05 |
| -192 | 116 | 20 | 29 | 30 | 0.05 |
| -207 | 127 | 20 | 30 | 30 | 0.05 |
| -222 | 139 | 20 | 30 | 30 | 0.05 |
| -235 | 151 | 19 | 31 | 30 | 0.05 |
| -248 | 164 | 18 | 32 | 30 | 0.05 |
| -261 | 178 | 17 | 32 | 30 | 0.05 |
| -272 | 193 | 17 | 32 | 30 | 0.05 |
| -283 | 208 | 17 | 33 | 30 | 0.05 |
| -293 | 224 | 17 | 34 | 30 | 0.05 |
| -303 | 239 | 17 | 34 | 30 | 0.05 |
| -312 | 256 | 17 | 35 | 30 | 0.05 |
| -320 | 272 | 17 | 36 | 30 | 0.05 |

Fig. 1. Averaged geometry area of comfort (AoC) generated from motion capture measurement with confidence intervals (data based on value from table 1)

Table 2. List of points in the area of approval (AoA). Where: x and y - location of the control point on the sagittal plane, δx - confidence interval of the control point location on the horizontal axis, δy - confidence interval of the control point location on the vertical axis, n - the size of the sample used to calculate the control point position, p - adopted confidence level.

| x | y | δx | δy | n | p |
| --- | --- | --- | --- | --- | --- |
| *[mm]* | *[mm]* | *[mm]* | *[mm]* | *n/a* | *n/a* |
| -16 | 11 | 13 | 16 | 30 | 0.05 |
| -41 | 9 | 13 | 17 | 30 | 0.05 |
| -68 | 7 | 14 | 17 | 30 | 0.05 |
| -94 | 6 | 16 | 16 | 30 | 0.05 |
| -120 | 6 | 18 | 14 | 30 | 0.05 |
| -146 | 6 | 20 | 14 | 30 | 0.05 |
| -172 | 7 | 22 | 14 | 30 | 0.05 |
| -198 | 10 | 24 | 14 | 30 | 0.05 |
| -223 | 13 | 27 | 16 | 30 | 0.05 |
| -249 | 16 | 30 | 17 | 30 | 0.05 |
| -275 | 20 | 32 | 18 | 30 | 0.05 |
| -300 | 25 | 35 | 20 | 30 | 0.05 |
| -326 | 32 | 37 | 20 | 30 | 0.05 |
| -350 | 40 | 41 | 22 | 30 | 0.05 |
| -373 | 51 | 43 | 22 | 30 | 0.05 |
| -396 | 63 | 46 | 21 | 30 | 0.05 |
| -419 | 76 | 48 | 21 | 30 | 0.05 |
| -442 | 88 | 51 | 21 | 30 | 0.05 |
| -465 | 101 | 53 | 21 | 30 | 0.05 |
| -486 | 116 | 55 | 21 | 30 | 0.05 |
| -508 | 130 | 57 | 21 | 30 | 0.05 |
| -529 | 145 | 58 | 21 | 30 | 0.05 |
| -550 | 161 | 60 | 22 | 30 | 0.05 |
| -569 | 178 | 62 | 21 | 30 | 0.05 |
| -589 | 196 | 64 | 21 | 30 | 0.05 |
| -608 | 213 | 67 | 21 | 30 | 0.05 |
| -626 | 231 | 70 | 21 | 30 | 0.05 |
| -645 | 250 | 73 | 20 | 30 | 0.05 |
| -661 | 270 | 75 | 19 | 30 | 0.05 |
| -678 | 290 | 78 | 19 | 30 | 0.05 |
| -693 | 311 | 81 | 20 | 30 | 0.05 |
| -708 | 333 | 82 | 21 | 30 | 0.05 |
| -722 | 355 | 84 | 22 | 30 | 0.05 |
| -734 | 377 | 85 | 23 | 30 | 0.05 |
| -746 | 400 | 87 | 24 | 30 | 0.05 |
| -754 | 423 | 85 | 25 | 30 | 0.05 |
| -753 | 446 | 73 | 27 | 30 | 0.05 |
| -748 | 467 | 62 | 25 | 30 | 0.05 |
| -740 | 487 | 53 | 22 | 30 | 0.05 |
| -727 | 502 | 53 | 21 | 30 | 0.05 |
| -712 | 516 | 59 | 23 | 30 | 0.05 |
| -688 | 523 | 59 | 23 | 30 | 0.05 |
| -662 | 527 | 59 | 23 | 30 | 0.05 |
| -636 | 531 | 58 | 24 | 30 | 0.05 |
| -610 | 535 | 58 | 24 | 30 | 0.05 |
| -584 | 538 | 58 | 26 | 30 | 0.05 |
| -558 | 541 | 58 | 27 | 30 | 0.05 |
| -532 | 544 | 58 | 29 | 30 | 0.05 |
| -507 | 547 | 58 | 31 | 30 | 0.05 |
| -480 | 548 | 58 | 30 | 30 | 0.05 |
| -454 | 550 | 59 | 29 | 30 | 0.05 |
| -428 | 552 | 59 | 28 | 30 | 0.05 |
| -402 | 553 | 60 | 27 | 30 | 0.05 |
| -376 | 555 | 61 | 26 | 30 | 0.05 |
| -350 | 556 | 62 | 26 | 30 | 0.05 |
| -324 | 557 | 63 | 26 | 30 | 0.05 |
| -298 | 558 | 64 | 27 | 30 | 0.05 |
| -272 | 559 | 65 | 27 | 30 | 0.05 |
| -246 | 559 | 67 | 26 | 30 | 0.05 |
| -220 | 559 | 68 | 25 | 30 | 0.05 |
| -194 | 558 | 69 | 24 | 30 | 0.05 |
| -168 | 558 | 71 | 24 | 30 | 0.05 |
| -142 | 557 | 73 | 24 | 30 | 0.05 |
| -116 | 556 | 74 | 24 | 30 | 0.05 |
| -90 | 555 | 76 | 24 | 30 | 0.05 |
| -64 | 553 | 78 | 23 | 30 | 0.05 |
| -38 | 551 | 80 | 22 | 30 | 0.05 |
| -12 | 547 | 82 | 21 | 30 | 0.05 |
| 11 | 538 | 85 | 22 | 30 | 0.05 |
| 34 | 526 | 89 | 24 | 30 | 0.05 |
| 56 | 513 | 95 | 26 | 30 | 0.05 |
| 77 | 499 | 100 | 28 | 30 | 0.05 |
| 99 | 485 | 105 | 31 | 30 | 0.05 |
| 119 | 470 | 110 | 35 | 30 | 0.05 |
| 138 | 453 | 114 | 38 | 30 | 0.05 |
| 154 | 434 | 116 | 40 | 30 | 0.05 |
| 169 | 413 | 115 | 40 | 30 | 0.05 |
| 181 | 390 | 113 | 38 | 30 | 0.05 |
| 193 | 367 | 111 | 37 | 30 | 0.05 |
| 204 | 344 | 109 | 36 | 30 | 0.05 |
| 214 | 321 | 107 | 34 | 30 | 0.05 |
| 224 | 297 | 105 | 32 | 30 | 0.05 |
| 234 | 273 | 104 | 31 | 30 | 0.05 |
| 241 | 249 | 103 | 31 | 30 | 0.05 |
| 247 | 224 | 100 | 31 | 30 | 0.05 |
| 252 | 199 | 97 | 31 | 30 | 0.05 |
| 255 | 174 | 92 | 32 | 30 | 0.05 |
| 252 | 150 | 83 | 31 | 30 | 0.05 |
| 244 | 129 | 69 | 30 | 30 | 0.05 |
| 234 | 109 | 55 | 29 | 30 | 0.05 |
| 223 | 90 | 40 | 29 | 30 | 0.05 |
| 207 | 73 | 31 | 30 | 30 | 0.05 |
| 184 | 62 | 27 | 24 | 30 | 0.05 |
| 161 | 53 | 23 | 20 | 30 | 0.05 |
| 137 | 44 | 21 | 16 | 30 | 0.05 |
| 112 | 36 | 18 | 14 | 30 | 0.05 |
| 87 | 29 | 17 | 12 | 30 | 0.05 |
| 62 | 22 | 15 | 12 | 30 | 0.05 |
| 36 | 18 | 14 | 13 | 30 | 0.05 |
| 10 | 15 | 13 | 14 | 30 | 0.05 |
| -16 | 11 | 13 | 16 | 30 | 0.05 |
| -16 | 11 | 13 | 16 | 30 | 0.05 |
| -41 | 9 | 13 | 17 | 30 | 0.05 |
| -68 | 7 | 14 | 17 | 30 | 0.05 |
| -94 | 6 | 16 | 16 | 30 | 0.05 |
| -120 | 6 | 18 | 14 | 30 | 0.05 |
| -146 | 6 | 20 | 14 | 30 | 0.05 |
| -172 | 7 | 22 | 14 | 30 | 0.05 |
| -198 | 10 | 24 | 14 | 30 | 0.05 |
| -223 | 13 | 27 | 16 | 30 | 0.05 |
| -249 | 16 | 30 | 17 | 30 | 0.05 |
| -275 | 20 | 32 | 18 | 30 | 0.05 |
| -300 | 25 | 35 | 20 | 30 | 0.05 |
| -326 | 32 | 37 | 20 | 30 | 0.05 |
| -350 | 40 | 41 | 22 | 30 | 0.05 |
| -373 | 51 | 43 | 22 | 30 | 0.05 |
| -396 | 63 | 46 | 21 | 30 | 0.05 |
| -419 | 76 | 48 | 21 | 30 | 0.05 |
| -442 | 88 | 51 | 21 | 30 | 0.05 |
| -465 | 101 | 53 | 21 | 30 | 0.05 |
| -486 | 116 | 55 | 21 | 30 | 0.05 |
| -508 | 130 | 57 | 21 | 30 | 0.05 |
| -529 | 145 | 58 | 21 | 30 | 0.05 |
| -550 | 161 | 60 | 22 | 30 | 0.05 |
| -569 | 178 | 62 | 21 | 30 | 0.05 |

Fig. 2. Averaged geometry area of approval (AoA) generated from motion capture measurement with confidence intervals (data based on value from table 2)

Table 3. List of points in the area of risk (AoR). Where: x and y - location of the control point on the sagittal plane, δx - confidence interval of the control point location on the horizontal axis, δy - confidence interval of the control point location on the vertical axis, n - the size of the sample used to calculate the control point position, p - adopted confidence level.

| x | y | δx | δy | n | p |
| --- | --- | --- | --- | --- | --- |
| *[mm]* | *[mm]* | *[mm]* | *[mm]* | *n/a* | *n/a* |
| -5 | -123 | 19 | 63 | 30 | 0.05 |
| 26 | -114 | 18 | 65 | 30 | 0.05 |
| 58 | -105 | 18 | 67 | 30 | 0.05 |
| 90 | -96 | 19 | 69 | 30 | 0.05 |
| 119 | -82 | 19 | 70 | 30 | 0.05 |
| 148 | -66 | 19 | 72 | 30 | 0.05 |
| 175 | -48 | 21 | 74 | 30 | 0.05 |
| 198 | -27 | 24 | 77 | 30 | 0.05 |
| 220 | -3 | 28 | 79 | 30 | 0.05 |
| 240 | 23 | 33 | 81 | 30 | 0.05 |
| 257 | 51 | 35 | 84 | 30 | 0.05 |
| 271 | 80 | 38 | 88 | 30 | 0.05 |
| 284 | 110 | 40 | 91 | 30 | 0.05 |
| 294 | 141 | 37 | 93 | 30 | 0.05 |
| 304 | 172 | 36 | 95 | 30 | 0.05 |
| 311 | 204 | 35 | 97 | 30 | 0.05 |
| 315 | 236 | 37 | 99 | 30 | 0.05 |
| 317 | 268 | 41 | 101 | 30 | 0.05 |
| 317 | 300 | 46 | 103 | 30 | 0.05 |
| 315 | 332 | 51 | 105 | 30 | 0.05 |
| 306 | 360 | 61 | 101 | 30 | 0.05 |
| 295 | 386 | 72 | 94 | 30 | 0.05 |
| 279 | 412 | 82 | 86 | 30 | 0.05 |
| 263 | 436 | 92 | 78 | 30 | 0.05 |
| 244 | 460 | 100 | 70 | 30 | 0.05 |
| 223 | 481 | 108 | 60 | 30 | 0.05 |
| 201 | 501 | 118 | 51 | 30 | 0.05 |
| 174 | 517 | 123 | 46 | 30 | 0.05 |
| 146 | 533 | 128 | 42 | 30 | 0.05 |
| 118 | 547 | 133 | 38 | 30 | 0.05 |
| 88 | 556 | 136 | 32 | 30 | 0.05 |
| 57 | 562 | 137 | 27 | 30 | 0.05 |
| 24 | 566 | 138 | 26 | 30 | 0.05 |
| -8 | 569 | 140 | 27 | 30 | 0.05 |
| -41 | 570 | 141 | 26 | 30 | 0.05 |
| -74 | 569 | 143 | 23 | 30 | 0.05 |
| -107 | 567 | 145 | 20 | 30 | 0.05 |
| -139 | 565 | 146 | 18 | 30 | 0.05 |
| -172 | 562 | 148 | 18 | 30 | 0.05 |
| -205 | 558 | 150 | 18 | 30 | 0.05 |
| -238 | 554 | 152 | 19 | 30 | 0.05 |
| -270 | 550 | 154 | 20 | 30 | 0.05 |
| -303 | 545 | 155 | 22 | 30 | 0.05 |
| -335 | 540 | 157 | 24 | 30 | 0.05 |
| -368 | 535 | 159 | 25 | 30 | 0.05 |
| -400 | 529 | 160 | 27 | 30 | 0.05 |
| -433 | 523 | 162 | 28 | 30 | 0.05 |
| -465 | 516 | 163 | 30 | 30 | 0.05 |
| -497 | 509 | 165 | 32 | 30 | 0.05 |
| -529 | 502 | 166 | 34 | 30 | 0.05 |
| -561 | 494 | 168 | 36 | 30 | 0.05 |
| -593 | 487 | 169 | 38 | 30 | 0.05 |
| -625 | 479 | 171 | 39 | 30 | 0.05 |
| -657 | 471 | 172 | 43 | 30 | 0.05 |
| -689 | 463 | 174 | 46 | 30 | 0.05 |
| -721 | 455 | 176 | 49 | 30 | 0.05 |
| -753 | 447 | 177 | 52 | 30 | 0.05 |
| -785 | 439 | 179 | 56 | 30 | 0.05 |
| -815 | 429 | 181 | 59 | 30 | 0.05 |
| -845 | 418 | 184 | 64 | 30 | 0.05 |
| -873 | 403 | 188 | 65 | 30 | 0.05 |
| -900 | 386 | 194 | 63 | 30 | 0.05 |
| -925 | 367 | 201 | 63 | 30 | 0.05 |
| -944 | 347 | 206 | 61 | 30 | 0.05 |
| -952 | 323 | 201 | 59 | 30 | 0.05 |
| -952 | 296 | 194 | 60 | 30 | 0.05 |
| -944 | 269 | 182 | 60 | 30 | 0.05 |
| -930 | 244 | 166 | 57 | 30 | 0.05 |
| -915 | 218 | 151 | 55 | 30 | 0.05 |
| -900 | 193 | 135 | 54 | 30 | 0.05 |
| -883 | 169 | 122 | 52 | 30 | 0.05 |
| -860 | 147 | 114 | 50 | 30 | 0.05 |
| -837 | 125 | 107 | 49 | 30 | 0.05 |
| -814 | 103 | 100 | 49 | 30 | 0.05 |
| -791 | 82 | 93 | 49 | 30 | 0.05 |
| -764 | 63 | 88 | 49 | 30 | 0.05 |
| -738 | 44 | 84 | 49 | 30 | 0.05 |
| -711 | 25 | 79 | 50 | 30 | 0.05 |
| -684 | 7 | 75 | 50 | 30 | 0.05 |
| -656 | -10 | 72 | 49 | 30 | 0.05 |
| -628 | -27 | 69 | 49 | 30 | 0.05 |
| -600 | -44 | 66 | 50 | 30 | 0.05 |
| -572 | -60 | 64 | 51 | 30 | 0.05 |
| -542 | -74 | 61 | 49 | 30 | 0.05 |
| -513 | -88 | 59 | 49 | 30 | 0.05 |
| -483 | -100 | 56 | 50 | 30 | 0.05 |
| -453 | -112 | 53 | 52 | 30 | 0.05 |
| -422 | -121 | 50 | 50 | 30 | 0.05 |
| -390 | -130 | 46 | 50 | 30 | 0.05 |
| -359 | -138 | 43 | 49 | 30 | 0.05 |
| -327 | -146 | 40 | 49 | 30 | 0.05 |
| -295 | -154 | 37 | 49 | 30 | 0.05 |
| -262 | -159 | 34 | 48 | 30 | 0.05 |
| -230 | -161 | 31 | 46 | 30 | 0.05 |
| -198 | -161 | 28 | 45 | 30 | 0.05 |
| -165 | -158 | 26 | 44 | 30 | 0.05 |
| -133 | -153 | 24 | 48 | 30 | 0.05 |
| -101 | -147 | 22 | 53 | 30 | 0.05 |
| -69 | -139 | 21 | 57 | 30 | 0.05 |
| -37 | -131 | 20 | 60 | 30 | 0.05 |
| -5 | -123 | 19 | 63 | 30 | 0.05 |
| -5 | -123 | 19 | 63 | 30 | 0.05 |
| 26 | -114 | 18 | 65 | 30 | 0.05 |
| 58 | -105 | 18 | 67 | 30 | 0.05 |
| 90 | -96 | 19 | 69 | 30 | 0.05 |
| 119 | -82 | 19 | 70 | 30 | 0.05 |
| 148 | -66 | 19 | 72 | 30 | 0.05 |
| 175 | -48 | 21 | 74 | 30 | 0.05 |
| 198 | -27 | 24 | 77 | 30 | 0.05 |
| 220 | -3 | 28 | 79 | 30 | 0.05 |
| 240 | 23 | 33 | 81 | 30 | 0.05 |
| 257 | 51 | 35 | 84 | 30 | 0.05 |
| 271 | 80 | 38 | 88 | 30 | 0.05 |
| 284 | 110 | 40 | 91 | 30 | 0.05 |
| 294 | 141 | 37 | 93 | 30 | 0.05 |
| 304 | 172 | 36 | 95 | 30 | 0.05 |
| 311 | 204 | 35 | 97 | 30 | 0.05 |
| 315 | 236 | 37 | 99 | 30 | 0.05 |
| 317 | 268 | 41 | 101 | 30 | 0.05 |
| 317 | 300 | 46 | 103 | 30 | 0.05 |
| 315 | 332 | 51 | 105 | 30 | 0.05 |
| 306 | 360 | 61 | 101 | 30 | 0.05 |
| 295 | 386 | 72 | 94 | 30 | 0.05 |
| 279 | 412 | 82 | 86 | 30 | 0.05 |
| 263 | 436 | 92 | 78 | 30 | 0.05 |

Fig. 3. Averaged geometry area of risk (AoR) generated from motion capture measurement with confidence intervals (data based on value from table 3)

Table 4. List of points in the area of propulsion (AoP). Where: x and y - location of the control point on the sagittal plane, δx - confidence interval of the control point location on the horizontal axis, δy - confidence interval of the control point location on the vertical axis, n - the size of the sample used to calculate the control point position, p - adopted confidence level.

| x | y | δx | δy | n | p |
| --- | --- | --- | --- | --- | --- |
| *[mm]* | *[mm]* | *[mm]* | *[mm]* | *n/a* | *n/a* |
| -120 | 216 | 22 | 26 | 30 | 0.05 |
| -130 | 210 | 22 | 27 | 30 | 0.05 |
| -141 | 204 | 21 | 27 | 30 | 0.05 |
| -152 | 198 | 21 | 28 | 30 | 0.05 |
| -162 | 191 | 21 | 29 | 30 | 0.05 |
| -173 | 185 | 21 | 30 | 30 | 0.05 |
| -184 | 178 | 21 | 30 | 30 | 0.05 |
| -194 | 172 | 20 | 30 | 30 | 0.05 |
| -204 | 165 | 20 | 30 | 30 | 0.05 |
| -214 | 157 | 20 | 31 | 30 | 0.05 |
| -223 | 150 | 20 | 31 | 30 | 0.05 |
| -233 | 143 | 20 | 31 | 30 | 0.05 |
| -242 | 135 | 19 | 32 | 30 | 0.05 |
| -250 | 128 | 19 | 32 | 30 | 0.05 |
| -259 | 121 | 18 | 32 | 30 | 0.05 |
| -268 | 114 | 18 | 32 | 30 | 0.05 |
| -276 | 107 | 17 | 32 | 30 | 0.05 |
| -283 | 102 | 17 | 31 | 30 | 0.05 |
| -291 | 96 | 16 | 31 | 30 | 0.05 |
| -299 | 92 | 16 | 30 | 30 | 0.05 |
| -307 | 88 | 16 | 29 | 30 | 0.05 |
| -313 | 86 | 16 | 28 | 30 | 0.05 |
| -319 | 86 | 16 | 26 | 30 | 0.05 |
| -324 | 87 | 15 | 25 | 30 | 0.05 |
| -326 | 90 | 15 | 23 | 30 | 0.05 |
| -327 | 95 | 14 | 22 | 30 | 0.05 |
| -326 | 102 | 13 | 20 | 30 | 0.05 |
| -325 | 108 | 12 | 19 | 30 | 0.05 |
| -322 | 114 | 12 | 18 | 30 | 0.05 |
| -319 | 121 | 12 | 18 | 30 | 0.05 |
| -315 | 127 | 12 | 18 | 30 | 0.05 |
| -312 | 134 | 12 | 19 | 30 | 0.05 |
| -308 | 141 | 13 | 20 | 30 | 0.05 |
| -303 | 148 | 15 | 21 | 30 | 0.05 |
| -299 | 155 | 16 | 23 | 30 | 0.05 |
| -293 | 163 | 18 | 24 | 30 | 0.05 |
| -288 | 172 | 20 | 24 | 30 | 0.05 |
| -282 | 181 | 21 | 24 | 30 | 0.05 |
| -275 | 190 | 23 | 24 | 30 | 0.05 |
| -268 | 199 | 24 | 24 | 30 | 0.05 |
| -259 | 208 | 25 | 23 | 30 | 0.05 |
| -250 | 216 | 26 | 23 | 30 | 0.05 |
| -241 | 225 | 27 | 23 | 30 | 0.05 |
| -232 | 232 | 28 | 22 | 30 | 0.05 |
| -222 | 240 | 29 | 22 | 30 | 0.05 |
| -212 | 247 | 30 | 22 | 30 | 0.05 |
| -202 | 254 | 31 | 21 | 30 | 0.05 |
| -191 | 260 | 31 | 21 | 30 | 0.05 |
| -180 | 266 | 32 | 20 | 30 | 0.05 |
| -169 | 272 | 33 | 20 | 30 | 0.05 |
| -158 | 278 | 34 | 19 | 30 | 0.05 |
| -147 | 283 | 35 | 19 | 30 | 0.05 |
| -136 | 289 | 35 | 18 | 30 | 0.05 |
| -125 | 294 | 36 | 18 | 30 | 0.05 |
| -113 | 299 | 37 | 17 | 30 | 0.05 |
| -102 | 303 | 38 | 17 | 30 | 0.05 |
| -90 | 307 | 39 | 16 | 30 | 0.05 |
| -79 | 310 | 39 | 15 | 30 | 0.05 |
| -68 | 314 | 39 | 14 | 30 | 0.05 |
| -57 | 317 | 39 | 14 | 30 | 0.05 |
| -47 | 321 | 39 | 13 | 30 | 0.05 |
| -36 | 324 | 40 | 12 | 30 | 0.05 |
| -25 | 326 | 40 | 12 | 30 | 0.05 |
| -14 | 328 | 40 | 11 | 30 | 0.05 |
| -3 | 329 | 41 | 10 | 30 | 0.05 |
| 9 | 330 | 41 | 10 | 30 | 0.05 |
| 19 | 331 | 42 | 10 | 30 | 0.05 |
| 30 | 331 | 42 | 9 | 30 | 0.05 |
| 39 | 331 | 42 | 9 | 30 | 0.05 |
| 49 | 331 | 42 | 9 | 30 | 0.05 |
| 58 | 331 | 42 | 8 | 30 | 0.05 |
| 66 | 331 | 42 | 8 | 30 | 0.05 |
| 73 | 330 | 41 | 8 | 30 | 0.05 |
| 79 | 328 | 41 | 8 | 30 | 0.05 |
| 84 | 326 | 41 | 8 | 30 | 0.05 |
| 85 | 324 | 40 | 8 | 30 | 0.05 |
| 85 | 321 | 39 | 7 | 30 | 0.05 |
| 83 | 318 | 37 | 7 | 30 | 0.05 |
| 78 | 315 | 35 | 8 | 30 | 0.05 |
| 72 | 312 | 33 | 8 | 30 | 0.05 |
| 66 | 309 | 31 | 9 | 30 | 0.05 |
| 60 | 307 | 29 | 10 | 30 | 0.05 |
| 53 | 304 | 28 | 11 | 30 | 0.05 |
| 45 | 302 | 28 | 12 | 30 | 0.05 |
| 38 | 298 | 28 | 12 | 30 | 0.05 |
| 29 | 295 | 28 | 13 | 30 | 0.05 |
| 21 | 291 | 29 | 14 | 30 | 0.05 |
| 13 | 287 | 30 | 15 | 30 | 0.05 |
| 3 | 282 | 31 | 15 | 30 | 0.05 |
| -7 | 277 | 31 | 16 | 30 | 0.05 |
| -18 | 272 | 31 | 17 | 30 | 0.05 |
| -29 | 267 | 30 | 18 | 30 | 0.05 |
| -40 | 262 | 30 | 19 | 30 | 0.05 |
| -50 | 256 | 29 | 20 | 30 | 0.05 |
| -61 | 251 | 28 | 21 | 30 | 0.05 |
| -71 | 246 | 27 | 22 | 30 | 0.05 |
| -81 | 240 | 26 | 22 | 30 | 0.05 |
| -90 | 235 | 24 | 23 | 30 | 0.05 |
| -100 | 229 | 23 | 24 | 30 | 0.05 |
| -110 | 222 | 23 | 25 | 30 | 0.05 |
| -120 | 216 | 22 | 26 | 30 | 0.05 |
| -120 | 216 | 22 | 26 | 30 | 0.05 |
| -130 | 210 | 22 | 27 | 30 | 0.05 |
| -141 | 204 | 21 | 27 | 30 | 0.05 |
| -152 | 198 | 21 | 28 | 30 | 0.05 |
| -162 | 191 | 21 | 29 | 30 | 0.05 |
| -173 | 185 | 21 | 30 | 30 | 0.05 |
| -184 | 178 | 21 | 30 | 30 | 0.05 |
| -194 | 172 | 20 | 30 | 30 | 0.05 |
| -204 | 165 | 20 | 30 | 30 | 0.05 |
| -214 | 157 | 20 | 31 | 30 | 0.05 |
| -223 | 150 | 20 | 31 | 30 | 0.05 |
| -233 | 143 | 20 | 31 | 30 | 0.05 |
| -242 | 135 | 19 | 32 | 30 | 0.05 |
| -250 | 128 | 19 | 32 | 30 | 0.05 |
| -259 | 121 | 18 | 32 | 30 | 0.05 |
| -268 | 114 | 18 | 32 | 30 | 0.05 |
| -276 | 107 | 17 | 32 | 30 | 0.05 |
| -283 | 102 | 17 | 31 | 30 | 0.05 |
| -291 | 96 | 16 | 31 | 30 | 0.05 |
| -299 | 92 | 16 | 30 | 30 | 0.05 |
| -307 | 88 | 16 | 29 | 30 | 0.05 |
| -313 | 86 | 16 | 28 | 30 | 0.05 |
| -319 | 86 | 16 | 26 | 30 | 0.05 |
| -324 | 87 | 15 | 25 | 30 | 0.05 |

Fig. 4. Averaged geometry area of propulsion (AoP) generated from motion capture measurement with confidence intervals (data based on value from table 4)
